# Upside down sulphate dynamics in a saline inland lake

**DOI:** 10.1038/s41598-022-27355-9

**Published:** 2023-02-21

**Authors:** Rosanna Margalef-Marti, Mathieu Sebilo, Aubin Thibault De Chanvalon, Pierre Anschutz, Céline Charbonnier, Béatrice Lauga, Ivan Gonzalez-Alvarez, Emmanuel Tessier, David Amouroux

**Affiliations:** 1 Université de Pau Et Des Pays de L’Adour, E2S UPPA, CNRS, IPREM, Pau, France; 2 Universitat de Barcelona, Barcelona, Spain; 3 Sorbonne Université, CNRS, IEES, Paris, France; 4 Univ. Bordeaux, CNRS, Bordeaux INP, EPOC, UMR 5805, 33600 Pessac, France

**Keywords:** Biogeochemistry, Stable isotope analysis

## Abstract

The sulphur cycle has a key role on the fate of nutrients through its several interconnected reactions. Although sulphur cycling in aquatic ecosystems has been thoroughly studied since the early 70’s, its characterisation in saline endorheic lakes still deserves further exploration. Gallocanta Lake (NE Spain) is an ephemeral saline inland lake whose main sulphate source is found on the lake bed minerals and leads to dissolved sulphate concentrations higher than those of seawater. An integrative study including geochemical and isotopic characterization of surface water, porewater and sediment has been performed to address how sulphur cycling is constrained by the geological background. In freshwater and marine environments, sulphate concentration decreases with depth are commonly associated with bacterial sulphate reduction (BSR). However, in Gallocanta Lake sulphate concentrations in porewater increase from 60 mM at the water–sediment interface to 230 mM at 25 cm depth. This extreme increase could be caused by dissolution of the sulphate rich mineral epsomite (MgSO_4_·7H_2_O). Sulphur isotopic data was used to validate this hypothesis and demonstrate the occurrence of BSR near the water–sediment interface. This dynamic prevents methane production and release from the anoxic sediment, which is advantageous in the current context of global warming. These results underline that geological context should be considered in future biogeochemical studies of inland lakes with higher potential availability of electron acceptors in the lake bed compared to the water column.

## Introduction

Biogeochemical processes in aquatic environments are investigated to understand the pathways by which essential compounds for life are circulated. The sulphur cycle has a key role on these flows given its many interconnected reactions to other nutrients such as carbon or iron, among others^1–3^. Although sulphur cycling in freshwater and marine systems has been studied for many decades and its main pathways have been thoroughly described and reviewed^1–4^, the potential for different reactions in saline inland lakes still deserves further exploration.

Sulphate (SO_4_^2−^) is one of the main electron acceptors in anoxic habitats. Bacterial sulphate reduction (BSR) involves the production of hydrogen sulphide (H_2_S) which can be either reoxidized back to SO_4_^2−^ or lead to the precipitation of secondary minerals such as pyrite^3,4^. Organic matter, iron species, oxygen and light availability have a key role in promoting certain processes over others (e.g. oxidation vs reduction or biotic vs abiotic reactions)^5–8^. BSR is the main microbial process that remineralizes and recycles organic matter in marine systems, such as euxinic basins and continental margin sediments, because SO_4_^2−^ is highly available with a mean concentration of 28 mM in seawater^2^. In contrast, SO_4_^2−^ concentrations are generally 2 to 3 orders of magnitude lower in freshwater environments, which restricts BSR in sediments as SO_4_^2−^ becomes rapidly depleted. Consequently, methanogenesis becomes the main anaerobic process of organic matter remineralization in freshwater sediments^4^. Previous studies in brackish to hypersaline lakes have shown that high salinities do not necessarily inhibit BSR or sulphide oxidation^9–13^. However, hot-spots of BSR activity and the extent to which the source of SO_4_^2−^ in different athalassic saline systems can come from the lake bed minerals or from groundwater instead of from surface water are poorly documented yet essential to predict methanogenesis potential in these ecosystems.

In ephemeral inland wetlands, variations in chemical and physical parameters are dependent on evaporation, rainfall or groundwater inflows and directly impact biogeochemical cycles^14,15^. The organic matter sources and the geological characteristics of the setting also play a key role. Coupled geochemical and isotopic characterisation of sulphur compounds in different parts of the lake including vertical profiles (i.e. surface water, porewater, sediment and groundwater), can provide further insight into sulphur cycling in these aquatic environments. This will be especially useful to anticipate biogeochemistry variations upon climate change as increasing temperatures can contribute to the salinization of freshwater lakes.

The saline lake Gallocanta (40°58′00″N, 1°29′50″W), located on a plateau within the Iberian Range at 990 m a.s.l, can be used as a model study site given its following particular characteristics. It is the largest and best-preserved endorheic saline lake in western Europe. It is shallow and has a pH ranging between 8 and 10^16^. Its maximum water depth for the last 30 years has remained below 1 m, occasionally with periods of complete dryness as the climate of the region is semiarid^16–18^. The water volume of the lake varies mainly due to the rates of evaporation and precipitation, which generate important runoff water flows and influences the groundwater table^16–19^. The groundwater flow tends towards the lake and discharges to it through detritic Quaternary material^19,20^. The multilayer aquifer system surrounding Gallocanta Lake is composed of an unconfined detritic Quaternary aquifer and a partially permeable Mesozoic carbonated aquifer^16,19,20^. The origin of the depression is karstic and overlies Triassic clays and evaporites (Keuper facies) although quaternary materials are found on the lake edges^21,22^. Mineralogy of the lake bed is rich in epsomite, hexahydrite, gypsum, quartz and phyllosilicates, halite, bischofite, calcite, dolomite and aragonite, whose proportion presents cross-shore and depth variations^23–25^. Previous studies on gypsum distribution in Gallocanta Lake sediment have shown spatial and depth variations. More specifically, gypsum amount increases from the shores to the centre of the lake. Furthermore, the maximum gypsum concentration is found between 70 and 100 cm depth in the shores of the lake while at the centre of the lake it is found at about 20 cm depth^23,25–29^. None of these studies report the spatial distribution of other SO_4_^2−^ minerals. Increases in salinity have been related to lower water volumes of the lake and during the dry periods when mainly carbonate and sulphate salts precipitate^23^. The goal of the present study was to study the occurrence of BSR and its main drivers in Gallocanta Lake using an integrated geochemical and isotopic approach.

## Saline endorheic Gallocanta Lake

**Figure 1:**
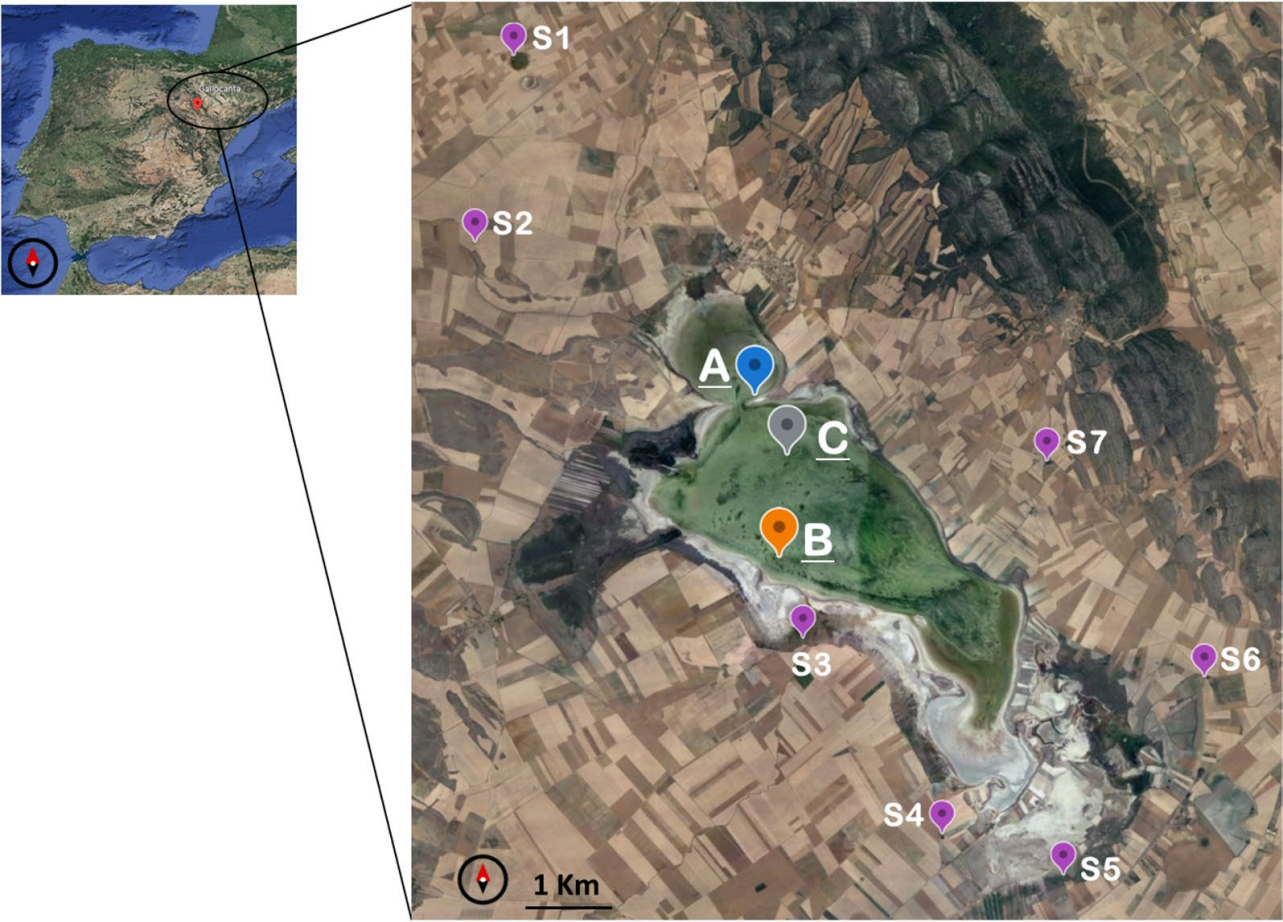
Study site. Sampling points in Gallocanta Lake (blue (A), orange (B) and grey (C)) and nearby sources (violet, S1 to S7). Image modified from Google Earth, ^©^ 2022.

Three sampling locations in the lake (A, B, C) were selected as representative areas of different cross-shore subenvironments to study sulphur cycling (Fig. 1). Surface water, porewater and sediment samples were collected in November 2020 (sites A and B) and June 2021 (sites A, B and C). These two seasons covered a daily temperature range from 6 to 14 °C and from 15 to 26 °C, respectively. Also, groundwater samples were obtained from available sources nearby the lake (Fig. 1). Conductivity measurements of surface water at sites A and B in November was 18.4 ± 1.6 mS/cm (Fig. S1). Calcium, magnesium, sodium and potassium concentrations were 15.5 ± 0.5, 75.5 ± 4.4, 240 ± 15 and 6.7 ± 0.7 mM, respectively (Fig. S2). The measured SO_4_^2−^ was 64.2 ± 2.2 mM (Fig. S3). The concentration of these major ions in surface water of Gallocanta Lake did not vary throughout the day but increased with depth in the water column (e.g. SO_4_^2−^ varied from 21 mM at the sub-surface to 37 mM in the bottom at 9:00 in site B on June). Furthermore, site C, which is closer to the centre of the lake, had a higher conductivity compared to A and B (45 ± 4 mS/cm). Chlorine and bicarbonate concentrations were not measured in this study but concentrations reported previously, considering cross-shore variations, reached up to 1890 and 14 mM, respectively^23,30^.

Detailed data on the hourly measurements of temperature, conductivity, dissolved O_2_, pH and organic carbon in surface water; major ions concentrations in surface and porewater; SO_4_^2−^ concentrations and isotopic composition in surface water, porewater, and groundwater; sulphide concentration in porewater; and bulk carbon content and isotopic composition in sediment is reported in the Supporting Information.

## Organic matter inputs

Gallocanta Lake is surrounded by vegetation and the lake bed is colonized by *Ruppia drepanensis,* which is a rooted submerged macrophyte commonly found in ephemeral saline inland wetlands of the Mediterranean region^31–33^. Also, it harbours a large community of migratory crane (*Grus grus*) from November to March each year^34–36^. Therefore, birds excrements and vegetation decay (mainly *R. drepanensis*) represent a significant source of organic matter and nutrients to the lake. Furthermore, daily measured dissolved O_2_ concentrations and pH values in surface water were highest at midday as a consequence of high rates of photosynthetic activity both in November and June, despite its salinity and alkalinity (e.g. pH increased from 9 at 11:00 to 9.8 at 15:00, in site B in November). Measured non-purgeable dissolved organic carbon (NPDOC) in surface water in November was 3.3 ± 0.03 mM for sites A and B and showed no daily variations. Nevertheless, the NPDOC concentration was higher in the bottom of the water column compared to the surface (e.g. 2.9 vs. 1.7 mM at 9:00 in site B in June, Fig. S4).

Bulk carbon detected in dry sediment ranged between 5.4 and 6.3% for site A, and between 6.8 and 9.0% for site B. In both cases the content decreased with sediment depth (up to 10 cm, Fig. S5a), which is consistent with mineralogy changes. Organic carbon content after decarbonation was 1% (site B, 4 cm), demonstrating the inorganic nature of the substrate with the exception of a layer rich in organic matter. This black layer is found immediately below the water–sediment interface (< 4 cm) and induces anoxic conditions. Previous studies in Gallocanta Lake also reported the highest organic carbon content on the first 10 cm of the sediment with values ranging from 1 to 6%^25,29,30^. The measured δ^13^C for bulk C in sediment ranged from − 7.5 to − 11.1 ‰ and showed an enrichment in the heavier isotopes with depth that coincides with the bulk C content decrease (Fig. S5b). δ^13^C values ranging from − 1.5 to − 11 ‰ have been previously reported for calcite, magnesite and dolomite in Gallocanta Lake sediments^24,37^. An increase on the heavy isotopes with depth was also observed in these studies. The δ^13^C measured for organic C was − 22.3 ‰ (site B, 4 cm). According to this result, the decreasing bulk C content accompanied by an increase in the δ^13^C values, points to a lower contribution of organic matter with depth^38^. According to this mass balance assumption, organic matter content is highest in the top layers of the sediment and the upper sediment has the higher mineralization potential.

## Sulphur cycling processes in a sulphate rich system

**Figure 2:**
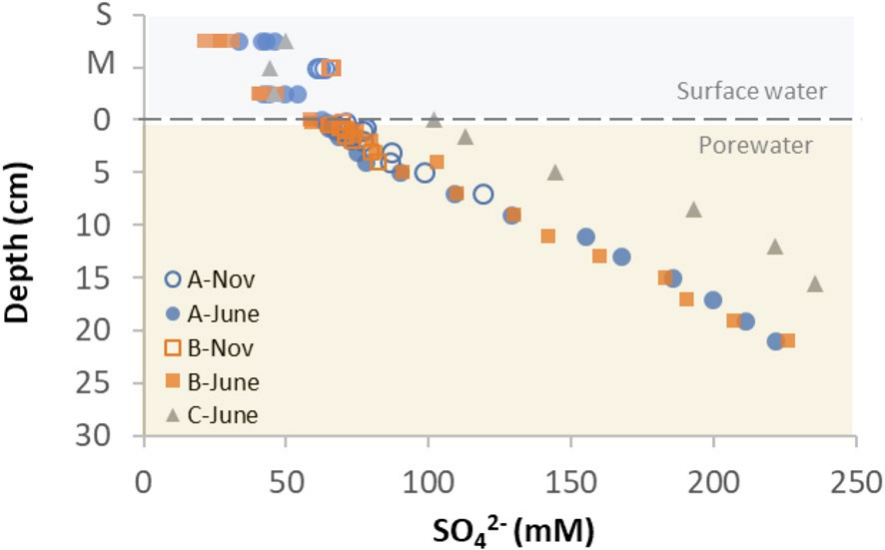
Sulphate concentration in surface and pore water. The water column depths ranged from 30 to 60 cm depending on the season and sampling point. In the plot, “*S*” = surface and “*M*” = middle. Deeper cores were obtained in June 2021 compared to November 2020.

The SO_4_^2−^ concentrations in the water column of Gallocanta Lake (21 to 65 mM) were similar or lower than those found for the upper layers of porewater (59 to 80 mM, < 2 cm depth). Below 2 cm, SO_4_^2−^ concentration increased with sediment depth to a maximum of 235 mM SO_4_^2−^ (sites A, B and C, Fig. 2). Sulphate concentrations were higher closer to the centre (site C) compared to the shore of the lake (sites A and B).

Sulphate concentration decreases in porewater are usually related to BSR, especially in anoxic environments with high organic matter inputs. Indeed, up to 600 µM for sulphide species (ΣS^2−^ = H_2_S + HS^−^ + S^2−^) were measured in the first mm of Gallocanta Lake sediment for site B and in a lesser extent for site A using a microprobe in November (Gonzalez-Álvarez et al., in preparation). The generation of sulphide in site B was confirmed during an additional sampling campaign performed in October 2022 (Fig. S6). Dissolved sulphide and iron sulphides such as framboidal pyrite, which can form in sediments containing ferrous iron and sulphide^26^, have been previously detected in Gallocanta Lake^25,26,30^. Also, in site B purple bacteria were observed in the water overlying the sediment cores immediately after collection, suggesting further sulphide oxidation on the water–sediment interface (Fig. S7). This is consistent with the low O_2_ concentrations measured at the bottom of the water column in site B in June (< 0.1 mM). Sulphate reducing and sulphur oxidizing microorganisms have been previously reported in bulk sediment samples from Gallocanta Lake^39^. However, although evidence of BSR and sulphide oxidation were robust, it is unlikely that these biological processes could produce almost a 200 mM increase over a 25 cm depth.

We hypothesized that SO_4_^2−^ concentration in porewater increased with depth due to SO_4_^2−^ dissolution from minerals or salts originating from the evaporites of the geological substrate. Also, that the most active location for BSR was the organic matter rich layer close to the water–sediment interface. Porewater concentration of Mg^2+^ correlate strongly with SO_4_^2−^ (r^2^ > 0.99, Fig. S8), and epsomite (MgSO_4_·7H_2_O) is highly undersaturated throughout the sediment (Saturation Index (SI) ranges from − 1.8 at depth to − 1 at the surface, based on Visual MINTEQ calculation) suggesting that dissolution of epsomite is the main source of SO_4_^2−^. Porewater Ca^2+^ concentrations are uniform with depth and the very low saturation of gypsum (SI ranges from 0.23 at depth to − 0.11 at the surface) indicate a possible equilibrium. Moreover, the much lower SO_4_^2−^ concentrations measured in groundwaters nearby the lake (4.7 mM, Figure S9), suggests that these sources are not contributing SO_4_^2−^ to the lake.

## δ^34^S data as a proxy to depict sulphate cycling pathways

The sulphur isotopic composition of SO_4_^2−^ (*δ*^34^S-SO_4_^2−^) can be used to trace its sources and transformation processes^40–42^. It has been established for some decades that BSR generate an isotopic fractionation leading to an increase of the *δ*^34^S values of the residual substrate in contrast to dilution that do not modify the isotopic signature^43^. Under closed system conditions, with no substrate renewal and in the absence of isotopic exchange, the isotopic fractionation (*ε*) is calculated by means of the Rayleigh distillation equation which involves the analyte concentration (C) and the determined isotopic composition:

$${\delta }_{residual} - {\delta }_{initial}= \varepsilon x Ln\left(\frac{{C}_{residual}}{{C}_{initial}}\right)$$

The use of the Rayleigh equation also implies a unidirectional and irreversible reaction. Instead, in open systems such as sediments influenced by mass exchange across the water–sediment interface and diffusive flows, the isotopic fractionation can be derived from the following equation proposed by Canfield (2001)^44^:

$${\delta }_{residual}= \frac{\left[\frac{{\delta }_{initial}}{\left(1-\frac{{C}_{residual}}{{C}_{initial}}\right)}\right]+\left[\frac{\varepsilon }{\left(\frac{\varepsilon }{1000}+1\right)}\right]}{\left[\frac{1}{\left(\frac{\varepsilon }{1000}+1\right)}\right]+\left[\frac{\left(\frac{{C}_{residual}}{{C}_{initial}}\right)}{\left(1-\frac{{C}_{residual}}{{C}_{initial}}\right)}\right]}$$

The *ε* values previously reported in the literature for BSR range between − 4 and − 66 ‰^43,45–50^. The main causes for these variations are related to the microbial SO_4_^2−^ metabolism and therefore to the SO_4_^2−^ reduction rates, the type and availability of electron donors, the temperature of the media and the active bacterial community^43,45–48^.

**Figure 3:**
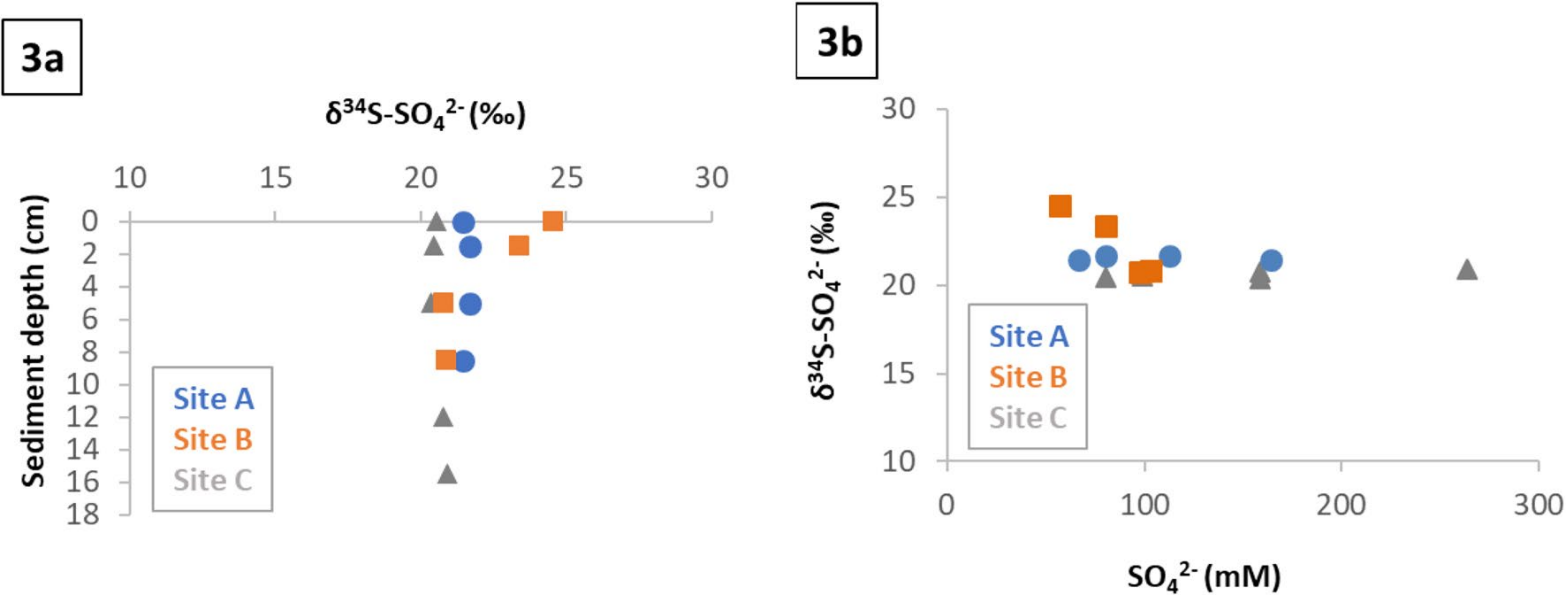
Porewater sulphate isotopic composition. Isotopic signature evolution with depth (**3a**) and with respect to concentration (**3b**). Data correspond to June 2021 samples.

The *δ*^34^S-SO_4_^2−^ values of surface water of Gallocanta were uniform across all sites, time, and season (+ 21.7 ± 0.3‰, Fig. S10). Also, no significant variation of *δ*^34^S-SO_4_^2−^ were observed in porewater of Gallocanta Lake from the middle to the bottom of the sediment cores collected in the sites A, B and C with an average of + 21.0 ± 0.5‰ (Fig. 3a). However, *δ*^34^S-SO_4_^2−^ increases up to + 24.5‰ in porewater in the site B from the middle to the top of the sediment core . This enrichment coincided with a decrease in SO_4_^2−^ concentrations, suggesting the process of BSR (Fig. 3b). The calculated *ε* value using the Rayleigh equation was − 6.6‰ (Fig. S11), which is in the range of those reported in the literature for BSR^43,45–48^. The use of this equation can be valid for Gallocanta sediment since the percentage of sulphur from SO_4_^2−^ reduction in the study site might be extremely low compared to the infinite amount of available substrate and because BSR rates can outcompete diffusive fluxes. To validate it, we compared the sample *δ*^34^S-SO_4_^2−^ values to the modelled trends with the open system equation. For this representation, we employed the determined *ɛ* values with Rayleigh and those estimated with the open system equation (Fig. S12). The obtained good fitting points that BSR in Gallocanta operates as in closed conditions, showing low or negligible effect from diffusion or advection processes on the SO_4_^2−^ isotopic fractionation.

**Figure 4:**
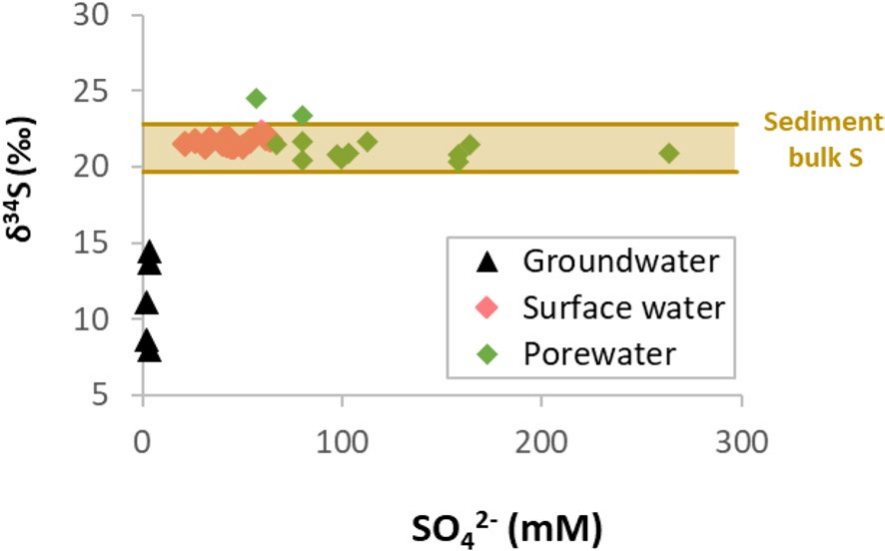
Sulphate isotopic composition versus concentration in water samples from Gallocanta Lake. The average δ^34^S of bulk S in sediment, including standard deviation, is presented as an ochre horizontal bar (bulk S content in sediment was 0.3–0.9%, which is not reflected in this figure).

The δ^34^S determined for bulk S in sediment (Site B, 9 and 21 cm depth) was + 21.2 ± 1.6‰, which is extremely close to the measured average δ^34^S-SO_4_^2−^ in surface water (+ 21.7 ± 0.3‰) and porewater (+ 21.0 ± 0.5‰). δ^34^S values reported for nearby Triassic evaporites (anhydrite) ranged from + 12.5 to + 14.5‰^51^. Precipitation of dissolved SO_4_^2−^ during gypsum formation can lead to an enrichment of heavy isotopes of up to 2‰^52^. Therefore, what was measured as bulk S seems to correspond to SO_4_^2−^ salts precipitated after several cycles of dissolution–precipitation of secondary SO_4_^2−^ minerals such as epsomite. On the other hand, significantly lower δ^34^S-SO_4_^2−^ values (from + 8.0 to + 14.5‰) were measured in groundwater (Fig. 4). Therefore, the lack of variations in the measured δ^34^S-SO_4_^2−^ for porewater samples in which concentration varied to almost 180 mM, elucidated that source of dissolved SO_4_^2−^ was the dissolution of local epsomite. In a similar study in the Salton Sea, SO_4_^2−^ dissolution from evaporite deposits and subsequent diffusion was observed but the possibility of BSR was not assessed^53^. Also, in the saline Devils lake (North Dakota), a bidirectional SO_4_^2−^ diffusion (from the lake bed and the water column) was observed at the BSR layer^11^. The SO_4_^2−^ concentration decreased from ~ 16 mM at the water–sediment interface to ~ 10 mM at 2 cm depth and then increased to ~ 24 mM at 8 cm depth^11^. Given the much high SO_4_^2−^ levels found in porewater compared to surface water of Gallocanta Lake, we only considered an upwards diffusion. Also, these high levels SO_4_^2−^ ensure BSR outcompetes methanogenesis.

**Figure 5:**
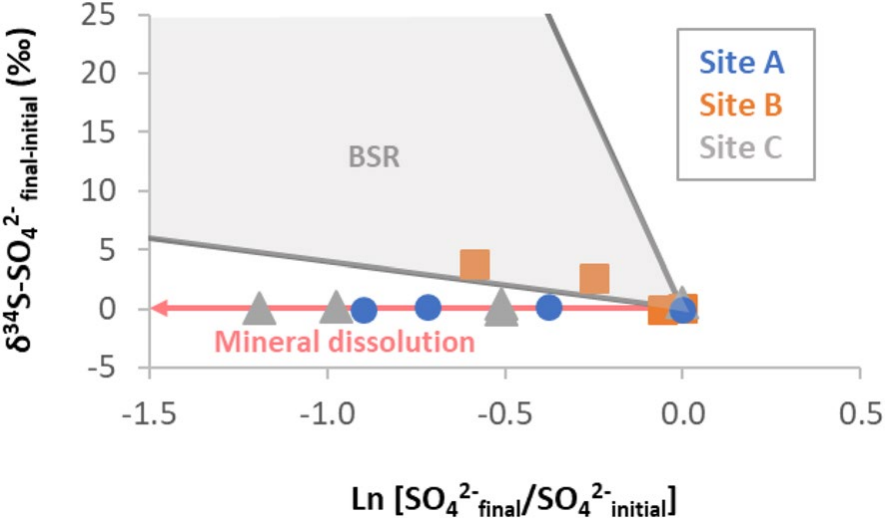
Sulphur cycling processes revealed by isotopic data. Theoretical trends for SO_4_^2−^ minerals/salts dissolution and BSR have been drawn by using ε = 0‰ and from − 4 to − 66‰, respectively. These lines are plotted together with the obtained results for our samples. For both axes, “final” corresponds to the values obtained at each depth while “initial” correspond to the deepest points (highest SO_4_^2−^ concentration).

A progressive decrease in SO_4_^2−^ concentrations from top to the bottom is commonly caused by BSR in oxygen depleted sediment in freshwater and marine environments^3,54^. Instead, in Gallocanta Lake SO_4_^2−^ concentrations increase with depth upon dissolution of SO_4_^2−^ rich minerals and salts. Sulphur isotopic data support this hypothesis and demonstrate that BSR is active in the organic rich layer below the water–sediment interface (Fig. 5). This was also supported by the high variations of SO_4_^2−^ concentrations, the detection of H_2_S and the observation of purple phototrophic bacteria. The sulphur cycling pathways observed in Gallocanta Lake could also be found in other systems containing soluble SO_4_^2−^ mineral beds and should be considered in studies aiming to determine the fate of nutrients. That is because in freshwater and marine environments, the main electron acceptors for the organic matter mineralization are available after diffusion from the water column (e.g. oxygen, nitrate or sulphate) or sedimentation (e.g. iron or manganese oxides). Contrarily, in saline inland lakes such as Gallocanta one of the major oxidants, SO_4_^2−^, can be available from the lake bed. This will likely constrain other nutrient cycling processes such as the methanogenesis.

## Concluding remarks

BSR in Gallocanta Lake occurs near the water–sediment interface. The source of SO_4_^2−^ in the system is found on the lake bed mineralogy and leads a 3 to 4 fold SO_4_^2−^ concentration increase in porewater with depth (reaching up to 235 mM). Continuously dissolved SO_4_^2−^ can be used as substrate for BSR and therefore, it prevents the occurrence of methanogenesis. The extremely high SO_4_^2−^ content in the system constrains not only sulphur cycling but also other biogeochemical processes. Coupling integrated geochemical and isotopic data from different lake compartments with field observations is valuable to understand ecosystem functioning.

## Materials and methods

The study involved two sampling campaigns to collect water and sediment samples from the study site for the subsequent chemical and isotopic characterization. The first was performed on November 2020 and the second on June 2021, to check seasonal variations. Two sampling points were stablished in the margins of the lake and named “A” and “B” and one closer to the centre of it “C”, to check spatial differences (Fig. 1). In November 2020, triplicate water samples were obtained from the middle of the water column following a diurnal cycle from sites A and B. In June 2021, water samples were obtained from two different water depths (subsurface and bottom of the water column) also following a diurnal cycle for sites A, B and C. In both campaigns, sediment cores were obtained early in the morning. Furthermore, water samples were also obtained from several sources identified nearby the lake during both sampling campaigns (Fig. 1). Details are reported in Table 1.

**Table 1:** Sampling campaigns.

	November 2020	June 2021
Sampled points (Fig. 1)	A + B	A + B + C
Timetable of water samples collection	Site A: 12, 14, 16 h	Sites A and B: 9, 11, 14, 16, 20 h
	Site B: 10, 12, 14, 16 h	Site C: 15 h
Sampled water column depths	Middle	Sub-surface, middle, bottom
Triplicates for water samples	Yes	No
Water depth	30–50 cm	40–60 cm
Timetable of sediment core collection	Site A: 12 h	Sites A and B: 9 h
	Site B: 10 h	Site C: 15 h
Triplicates for sediment cores	Yes	No
Sampled sources (Fig. 1)	S1 to S7	S5 to S7
Observations	Cranes present (26–28)	Rainfall events

Detailed information on the type of samples collected on each campaign.

Prior to each water sample collection, temperature, pH, Eh, O_2_ and conductivity were determined in situ by using a multiparametric probe (Aquaread AP-5000). Water samples were collected in 500 mL plastic flasks after three rinses. Collection at different water column depths was achieved by using 250 mL syringes connected to tygon tubes. Sediment cores were collected by using sealed PVC tubes (10 cm diameter, 40 cm height). Water and sediment samples were immediately treated after collection as follows.

An aliquot for each surface water sample was filtered through pre-ashed GFF filters and HCl acidified for NPDOC determination immediately after collection. The remaining water sample was filtered through 0.2 µm Sterivex Millipore^®^ filters. Aliquots for SO_4_^2−^ determination were HCl acidified while for dissolved major elements determination it was HNO_3_ acidified, they were all preserved at 4 °C. For the SO_4_^2−^ isotopic analysis, the dissolved SO_4_^2−^ was precipitated as BaSO_4_ by adding BaCl_2_·2H_2_O after acidifying the sample with HCl in order to prevent precipitation of BaCO_3_^55^.

Sediment cores were sliced inside an anaerobic chamber under a N_2_ atmosphere at different depth intervals, each slice was introduced into tubes and subsequently centrifuged to separate porewater from the solid fraction except for one aliquot that was preserved to determine the water content after lyophilization. After centrifugation, porewater samples were prepared with the same methods used for surface water samples. The solid fraction was frozen to determine the content and isotopic composition of C and S after lyophilization and milling in the laboratory.

The SO_4_^2−^ content was determined by nephelometry and DOC by organic matter combustion^56^. Concentration of major ions (Ca, Mg, K and Na) was determined by ICP-OES (iCAP 6000 series, Thermo Scientific). The amount of C and S in lyophilized sediment samples was measured with an elemental analyser (EA, Flash 2000, Thermo Scientific). The *δ*^34^S-SO_4_^2−^ was analysed with a Carlo Erba EA coupled in continuous flow to a Finnigan Delta XP Plus isotope ratio mass spectrometer (IRMS). The bulk carbon and sulphur content and isotopic composition (*δ*^13^C-C_bulk_ and *δ*^34^S-S_bulk_) of the sediment samples were determined by EA-IRMS (Flash 2000 EA and Delta V plus IRMS, Thermo Scientific). The content and *δ*^13^C of organic carbon in the sediment samples after decarbonation was also determined by EA-IRMS (Elementar-Isoprime)^57^. The standard deviation for *δ*^34^S and *δ*^13^C analyses was below ± 0.1‰. The isotopic notation is expressed in terms of *δ* per mil relative to international standards Vienna Canyon-Diabolo-Troilite (VCDT) for *δ*^34^S and Pee Dee Belemnite (VPDB) for *δ*^13^C, following:

$$\delta \, = \text{ } \frac{{\text{R}}_{\text{sample}}-{\text{R}}_{\text{standard}}}{{\text{R}}_{\text{standard}}},$$

where R = ^34^S/^32^S and ^13^C/^12^C, respectively.

## Supplementary Information

Supplementary Figures.

## Data Availability

The datasets generated during and/or analysed during the current study are available from the corresponding author on reasonable request.

## References

[1] Canfield, D. E.; Kristensen, E.; Thamdrup, B. The Sulfur Cycle. In *Advances in Marine Biology*; Aquatic Geomicrobiology; Academic Press, 2005; Vol. 48, pp 313–381. 10.1016/S0065-2881(05)48009-8. 10.1016/S0065-2881(05)48017-7 15797449

[2] Jørgensen BB, Findlay AJ, Pellerin A (2019). The biogeochemical sulfur cycle of marine sediments. Front. Microbiol..

[3] Thamdrup B, Fossing H, Jørgensen BB (1994). Manganese, iron and sulfur cycling in a coastal marine sediment, Aarhus Bay. Denmark. Geochim. Cosmochim. Acta.

[4] Holmer M, Storkholm P (2001). Sulphate reduction and sulphur cycling in lake sediments: A review. Freshw. Biol..

[5] Koschorreck M (2008). Microbial sulphate reduction at a low PH. FEMS Microbiol. Ecol..

[6] Kwon MJ, O’Loughlin EJ, Boyanov MI, Brulc JM, Johnston ER, Kemner KM, Antonopoulos DA (2016). Impact of organic carbon electron donors on microbial community development under iron- and sulfate-reducing conditions. PLoS ONE.

[7] Fründ C, Cohen Y (1992). Diurnal cycles of sulfate reduction under oxic conditions in cyanobacterial mats. Appl. Environ. Microbiol..

[8] Marschall C, Frenzel P, Cypionka H (1993). Influence of oxygen on sulfate reduction and growth of sulfate-reducing bacteria. Arch. Microbiol..

[9] Borzenko SV, Kolpakova MN, Shvartsev SL, Isupov VP (2018). Biogeochemical conversion of sulfur species in saline lakes of steppe Altai. J. Oceanol. Limnol..

[10] Häusler S, Weber M, Siebert C, Holtappels M, Noriega-Ortega BE, Beer DD, Ionescu D (2014). Sulfate reduction and sulfide oxidation in extremely steep salinity gradients formed by freshwater springs emerging into the dead sea. FEMS Microbiol Ecol.

[11] Komor SC (1992). Bidirectional sulfate diffusion in saline-lake sediments: Evidence from Devils Lake, Northeast North Dakota. Geology.

[12] Valiente N, Carrey R, Otero N, Gutiérrez-Villanueva MA, Soler A, Sanz D, Castaño S, Gómez-Alday JJ (2017). Tracing sulfate recycling in the hypersaline Pétrola Lake (SE Spain): A combined isotopic and microbiological approach. Chem. Geol..

[13] Moreira N, Walter L, Vasconcelos C, McKenzie J, McCall P (2004). Role of sulfide oxidation in dolomitization: Sediment and pore-water geochemistry of a modern hypersaline lagoon system. Geology.

[14] Jolly ID, McEwan KL, Holland KL (2008). A review of groundwater-surface water interactions in arid/semi-arid wetlands and the consequences of salinity for wetland ecology. Ecohydrology.

[15] Williams WD (2002). Environmental threats to salt lakes and the likely status of inland saline ecosystems in 2025. Environ. Conserv..

[16] CHE. *Confederación Hidrográfica del Ebro*. https://www.chebro.es/ (Accessed 1 June 2022).

[17] Comín FA, Rodó X, Comín P (1992). Lake Gallocanta (Aragon, NE Spain), a paradigm of fluctuations at different scales of time. Limnetica.

[18] Luna, E.; Latorre, B.; Castañeda, C. *Rainfall and the Presence of Water in Gallocanta Lake*. http://digital.csic.es/handle/10261/117417. (2014).

[19] San Roman Saldaña, J.; García Vera, M. Á.; Blasco Herguedas, Ó.; Coloma López, P. Toma de Datos, Modelación y Gestión Del Agua Subterránea En La Cuenca Endorréica de La Laguna de Gallocanta (España); Alicante, Spain, 2005; pp 551–557.

[20] Orellana-Macías JM, Merchán D, Causapé J (2020). Evolution and assessment of a nitrate vulnerable zone over 20 years: Gallocanta groundwater body (Spain). Hydrogeol. J..

[21] Gracia FJ, Gutierrez F, Gutierrez M (2002). Origin and evolution of the Gallocanta Polije (Iberian range, NE Spain). Z. Geomorph. N. F..

[22] García-Vera, M.A.; San Román Saldaña, J.; Blasco Herguedas, O.; Coloma López, P. Hidrogeología de La Laguna de GalIocanta e Implicaciones Ambientales. In M.A. Casterad and C. Castañeda (Eds.). La Laguna de Gallocanta: Medio Natural, Conservación y Teledetección. *Memorias de la Real Sociedad Española de Historia Natural.* **2009**, *7*, 79–104.

[23] Comín FA, Juli R, Comín P, Plana F (1990). Hydrogeochemistry of Lake Gallocanta (Aragón, NE Spain). Hydrobiologia.

[24] Mayayo MJ, Luzón A, Soria AR, Roc AC, Sánchez JA, Pérez A (2003). Sedimentological evolution of the holocene Gallocanta Lake, NE Spain. Limnol. Spain Tribute Kerry Kelts.

[25] Pérez A, Luzón A, Roc AC, Soria AR, Mayayo MJ, Sánchez JA (2002). Sedimentary facies distribution and genesis of a recent carbonate-rich Saline Lake: Gallocanta Lake, Iberian Chain, NE Spain. Sediment. Geol..

[26] Corzo A, Luzon A, Mayayo MJ, van Bergeijk SA, Mata P, de Lomas JG (2005). Carbonate mineralogy along a biogeochemical gradient in recent lacustrine sediments of Gallocanta Lake (Spain). Geomicrobiol. J..

[27] Castañeda C, Gracia FJ, Luna E, Rodríguez-Ochoa R (2015). Edaphic and geomorphic evidences of water level fluctuations in Gallocanta Lake, NE Spain. Geoderma.

[28] Luzón A, Pérez A, Mayayo MJ, Soria AR, Goñi MFS, Roc AC (2007). Holocene environmental changes in the Gallocanta lacustrine basin, Iberian range, NE Spain. Holocene.

[29] Schütt B (1998). Reconstruction of holocene paleoenvironments in the endorheic basin of laguna de Gallocanta, Central Spain by investigation of mineralogical and geochemical characters from lacustrine sediments. J. Paleolimnol..

[30] Castañeda C, Luna E, Rabenhorst M (2017). Reducing conditions in soil of Gallocanta Lake. Northeast Spain. Eur. J. Soil Sci..

[31] Castañeda C, Gracia FJ, Conesa JA, Latorre B (2020). Geomorphological control of habitat distribution in an intermittent shallow Saline Lake, Gallocanta Lake. NE Spain. Sci. Total Environ..

[32] Comín FA, Rodó X, Menéndez M (1993). Spatial heterogeneity of macrophytes in lake Gallocanta (Aragón, NE Spain). Hydrobiologia.

[33] Castro OD, Geraci A, Mannino AM, Mormile N, Santangelo A, Troia A (2021). A Contribution to the characterization of ruppia drepanensis (ruppiaceae), a key species of threatened mediterranean Wetlands. Ann. Mo. Bot. Gard..

[34] Alonso López JA, Alonso López JC, Cantos FJ, Bautista LM (1990). Spring crane grus grus migration through Gallocanta, Spain. II. Timing and pattern of daily departures. Ardea.

[35] Alonso López JC, Alonso López JA, Cantos FJ, Bautista LM (1990). Spring crane grus grus migration through Gallocanta, Spain. I. Daily Variations in Migration Volume. Ardea.

[36] Orellana-Macías JM, Bautista LM, Merchán D, Causapé J, Alonso JC (2020). Shifts in crane migration phenology associated with climate change in southwestern Europe. Avian Conserv. Ecol..

[37] Luzón A, Mayayo MJ, Pérez A (2009). Stable isotope characterisation of co-existing carbonates from the holocene Gallocanta Lake (NE Spain): Palaeolimnological implications. Int. J. Earth Sci..

[38] Accoe F, Boeckx P, Cleemput OV, Hofman G, Li hua R, Zhang Y, Guanxiong C (2002). Evolution of the Δ13C signature related to total carbon contents and carbon decomposition rate constants in a soil profile under grassland. Rapid Commun. Mass Spectrom..

[39] Menéndez-Serra M, Triadó-Margarit X, Castañeda C, Herrero J, Casamayor EO (2019). Microbial composition, potential functional roles and genetic novelty in gypsum-rich and hypersaline soils of Monegros and Gallocanta (Spain). Sci. Total Environ..

[40] Kendall C, McDonnell JJ (1999). Isotope Tracers in Catchment Hydrology.

[41] Mayer B, Fritz P, Prietzel J, Krouse HR (1995). The use of stable sulfur and oxygen isotope ratios for interpreting the mobility of sulfate in aerobic forest soils. Appl. Geochem..

[42] Otero N, Canals À, Soler A (2007). Using dual-isotope data to trace the origin and processes of dissolved sulphate: A case study in calders stream (Llobregat Basin, Spain). Aquat. Geochem..

[43] Canfield DE (2001). Isotope fractionation by natural populations of sulfate-reducing bacteria. Geochim. Cosmochim. Acta.

[44] Canfield DE (2001). Biogeochemistry of sulfur isotopes. Rev. Mineral. Geochem..

[45] Antler G, Turchyn AV, Ono S, Sivan O, Bosak T (2017). Combined 34S, 33S and 18O isotope fractionations record different intracellular steps of microbial sulfate reduction. Geochim. Cosmochim. Acta.

[46] Kaplan IR, Rittenberg SC (1964). Microbiological fractionation of sulphur isotopes. J. Gen. Microbiol..

[47] Mangalo M, Meckenstock RU, Stichler W, Einsiedl F (2007). Stable isotope fractionation during bacterial sulfate reduction is controlled by reoxidation of intermediates. Geochim. Cosmochim. Acta.

[48] Strebel O, Böttcher J, Fritz P (1990). Use of isotope fractionation of sulfate-sulfur and sulfate-oxygen to assess bacterial desulfurication in a sandy aquifer. J. Hydrol..

[49] Sim MS, Bosak T, Ono S (2011). Large sulfur isotope fractionation does not require disproportionation. Science.

[50] Leavitt WD, Halevy I, Bradley AS, Johnston DT (2013). Influence of sulfate reduction rates on the phanerozoic sulfur isotope record. Proc. Natl. Acad. Sci..

[51] Utrilla R, Pierre C, Orti F, Pueyo JJ (1992). Oxygen and sulphur isotope compositions as indicators of the origin of mesozoic and cenozoic evaporites from Spain. Chem. Geol..

[52] Driessche AESV, Canals A, Ossorio M, Reyes RC, García-Ruiz JM (2016). Unraveling the sulfate sources of (Giant) gypsum crystals using gypsum isotope fractionation factors. J. Geol..

[53] Wardlaw GD, Valentine DL (2005). Evidence for salt diffusion from sediments contributing to increasing salinity in the Salton sea, California. Hydrobiologia.

[54] Bak F, Pfennig N (1991). Microbial sulfate reduction in littoral sediment of lake constance. FEMS Microbiol. Lett..

[55] Dogramaci SS, Herczeg AL, Schiff SL, Bone Y (2001). Controls on Δ34S and Δ18O of dissolved sulfate in aquifers of the murray basin, Australia and their use as indicators of flow processes. Appl. Geochem..

[56] Rodier. *L’analyse de l’eau, eaux naturelles, eaux résiduaires, eau de mer*; Dunod, 1976.

[57] Romain, T. Tester Les Isotopes Stables de l’azote Des Matières Organiques Fossiles Terrestres Comme Marqueur Paléoclimatique Sur Des Séries Pré-Quaternaires, Université Pierre et Marie Curie - Paris VI, 2015. https://tel.archives-ouvertes.fr/tel-01408071.

