# Supplementary material for: Upside down sulphate dynamics in a saline inland lake
Source: Sci Rep. 2023 Feb 21;13:3032. doi: 10.1038/s41598-022-27355-9 (PMC9944303; doi:10.1038/s41598-022-27355-9)
Supplement: Supplementary file 1 — Supplementary Figures. [file 41598_2022_27355_MOESM1_ESM.docx]

# Supporting information

**Upside down sulphate dynamics in a saline inland lake**

Rosanna Margalef-Marti^1,2,*^, Mathieu Sebilo^3^, Aubin Thibault De Chanvalon^1^, Pierre Anschutz^4,^ Céline Charbonnier^4^, Béatrice Lauga^1^, Ivan Gonzalez-Alvarez^1^, Emmanuel Tessier^1^, David Amouroux^1^

*^1^ Université de Pau et des Pays de l’Adour, E2S UPPA, CNRS, IPREM, Pau, France; ^2^ Universitat de Barcelona, Barcelona, Spain; ^3^ Sorbonne Université, CNRS, IEES, Paris, France; ^4^ Univ. Bordeaux, CNRS, Bordeaux INP, EPOC, UMR 5805, F-33600 Pessac, France;* [**rosanna.margalef-marti@univ-pau.fr*](mailto:*rosanna.margalef-marti@univ-pau.fr)*;* [*rosannamargalef@ub.edu*](mailto:rosannamargalef@ub.edu)


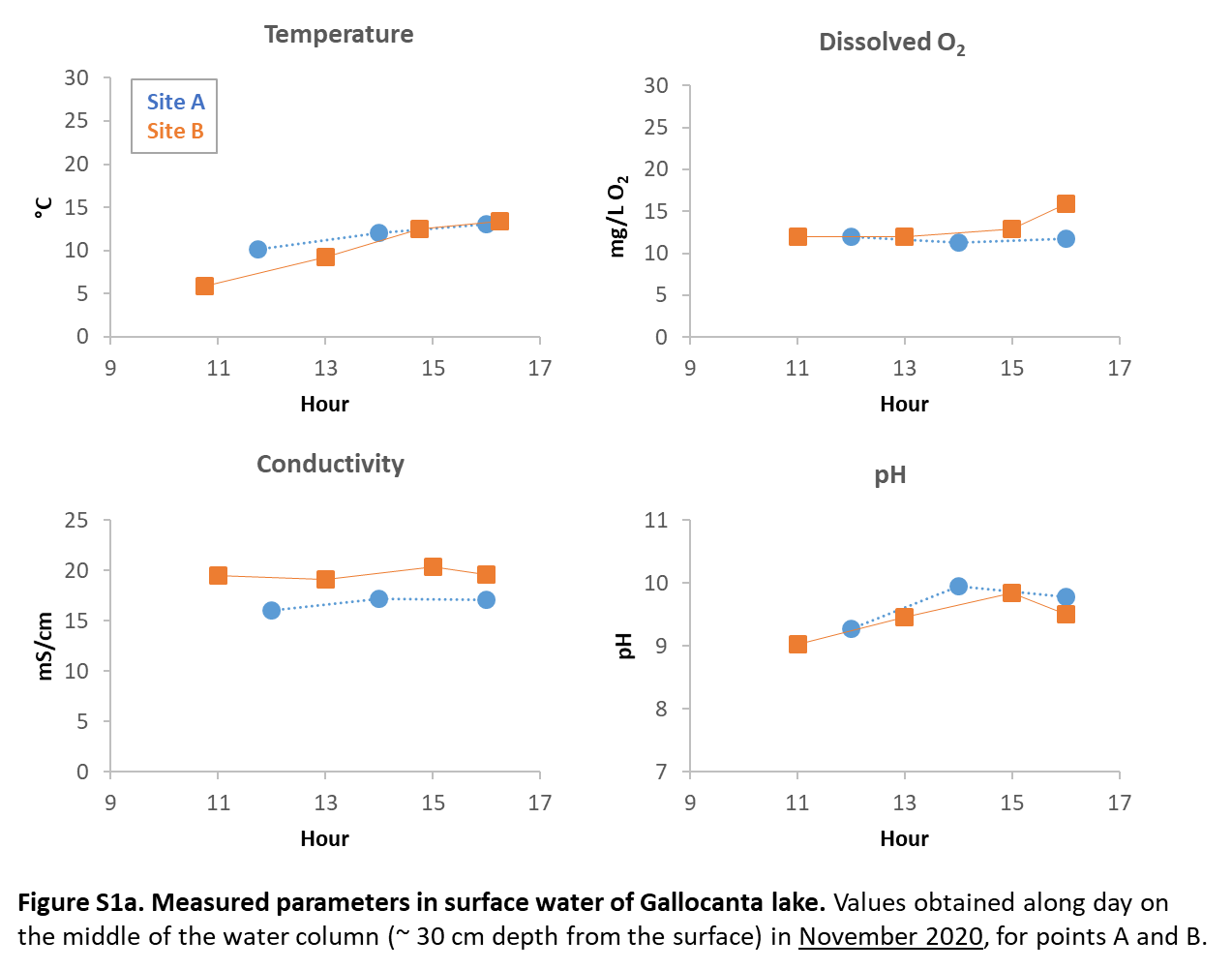


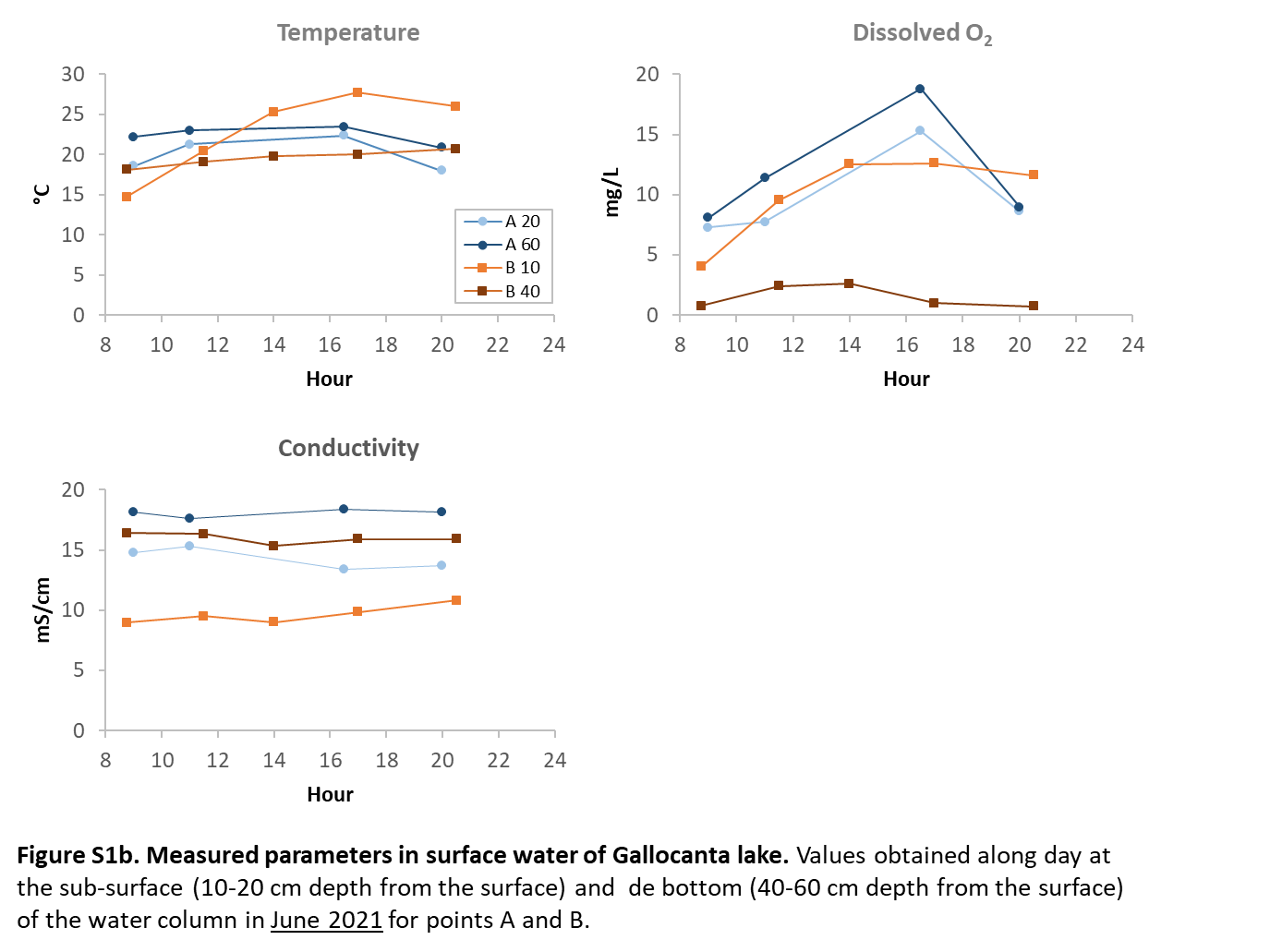


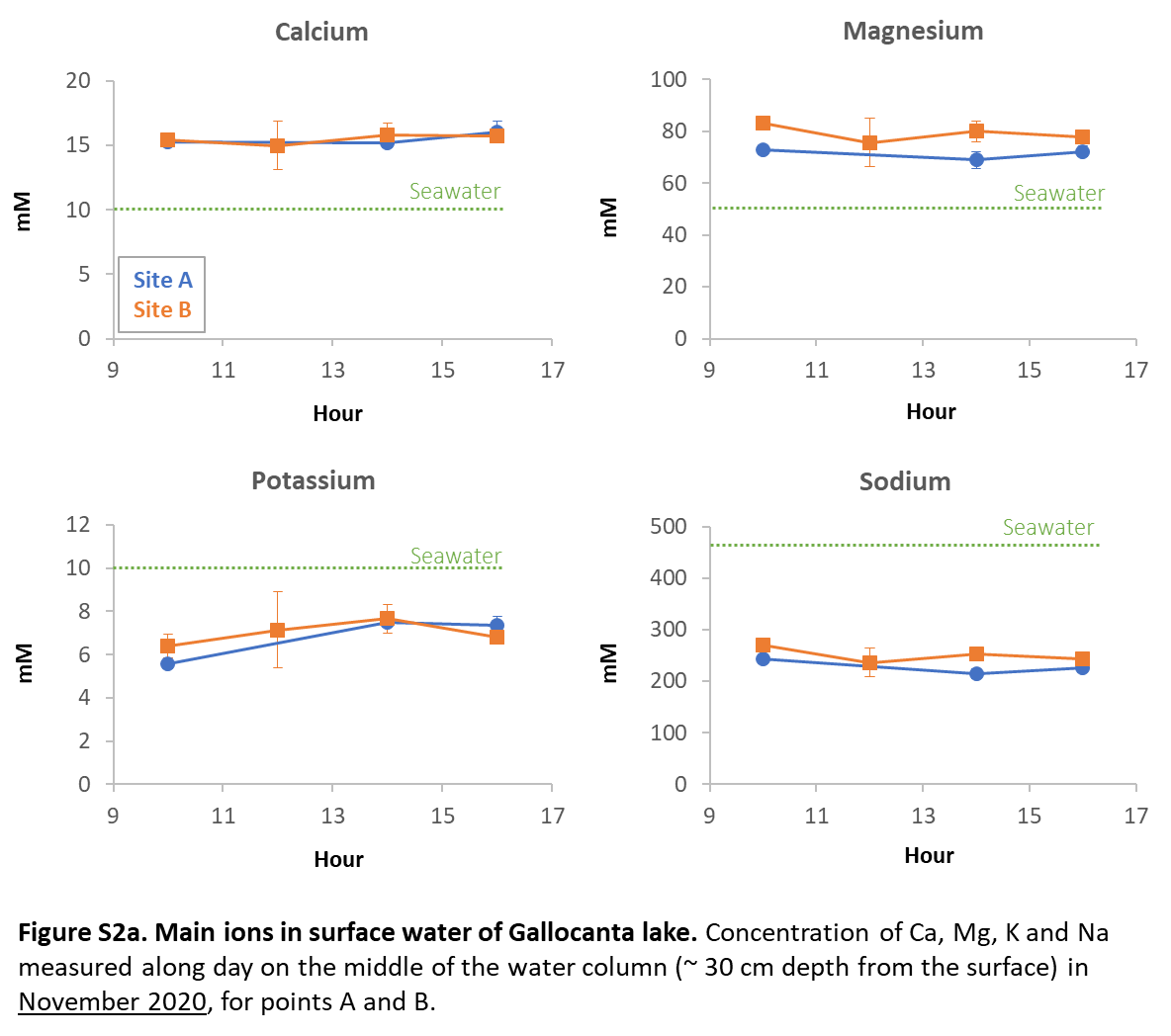


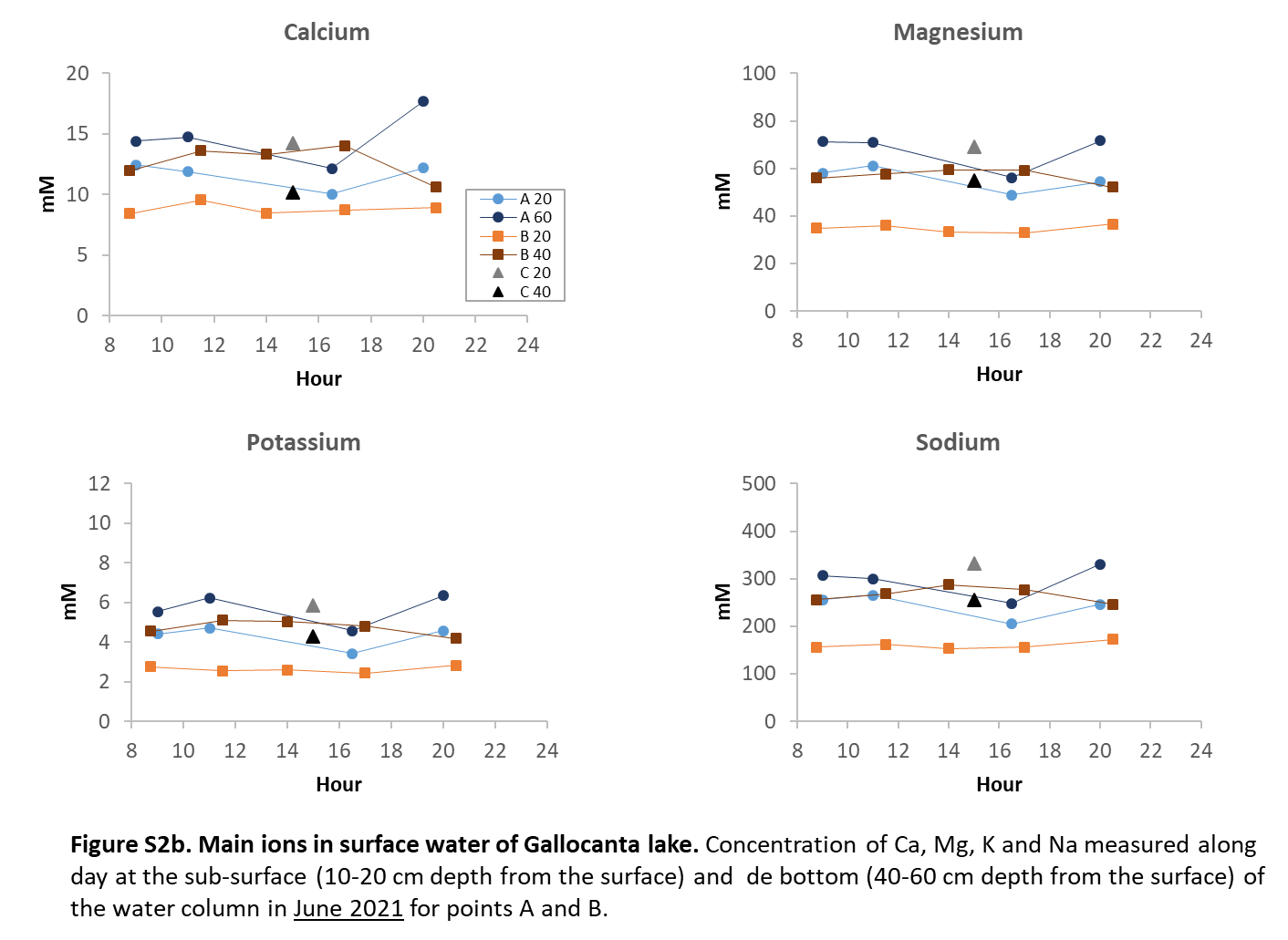


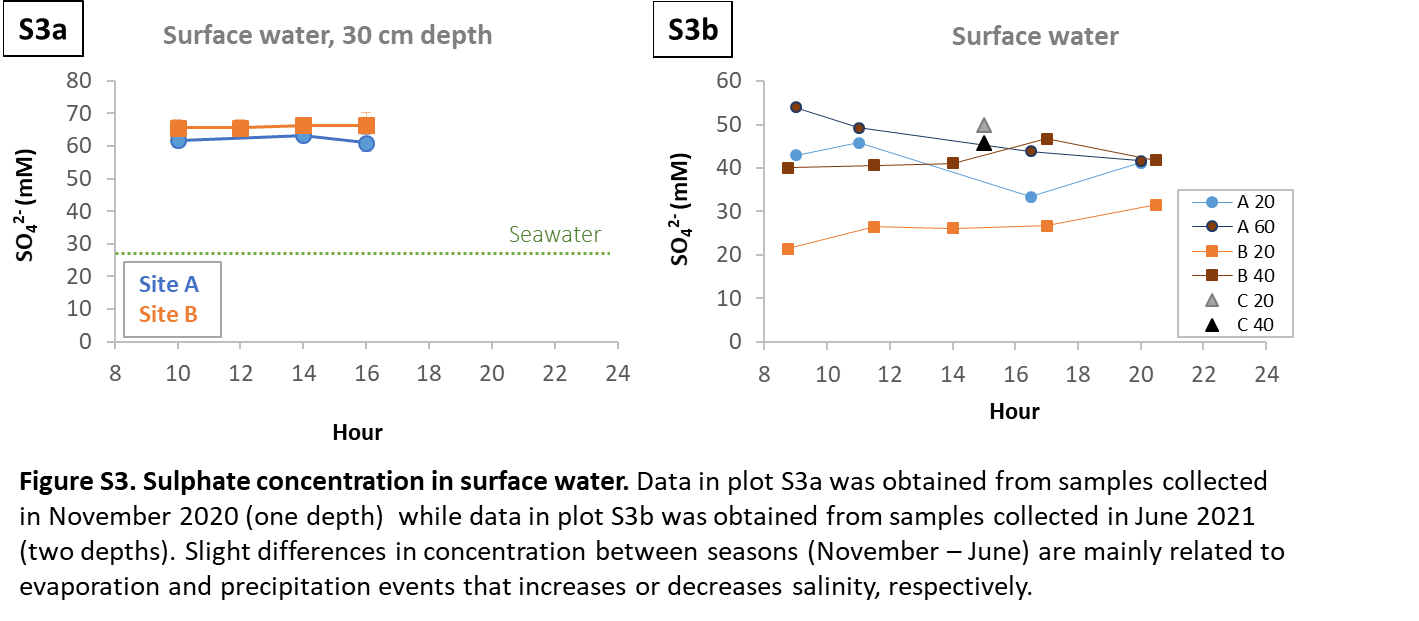


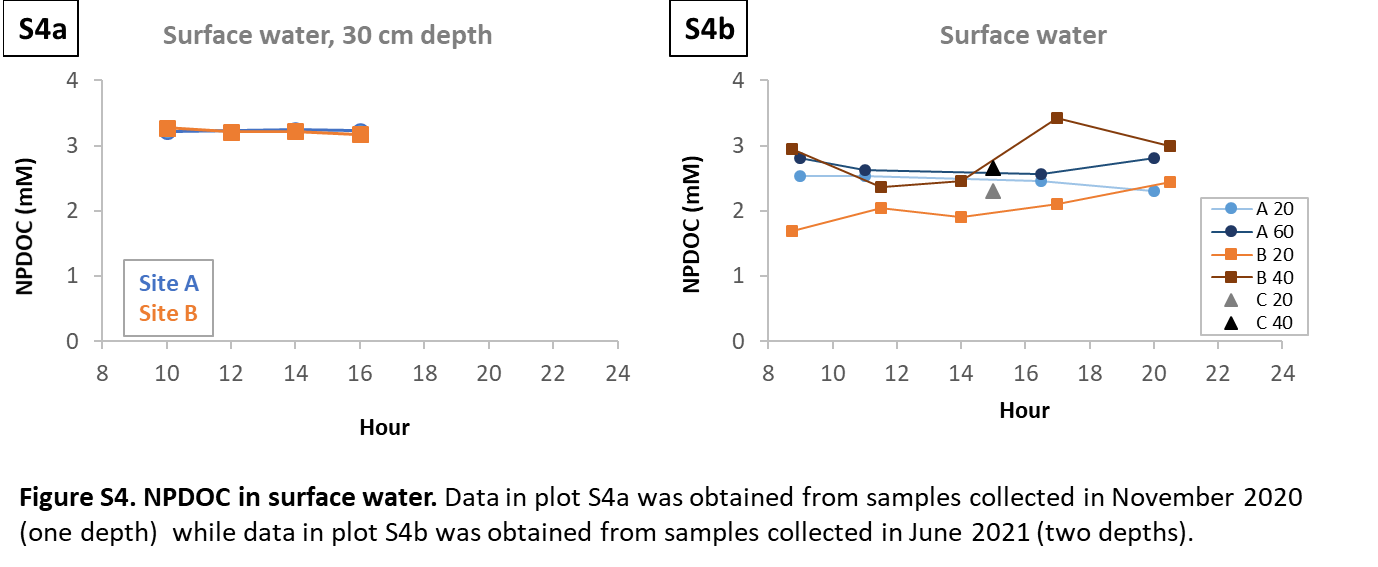


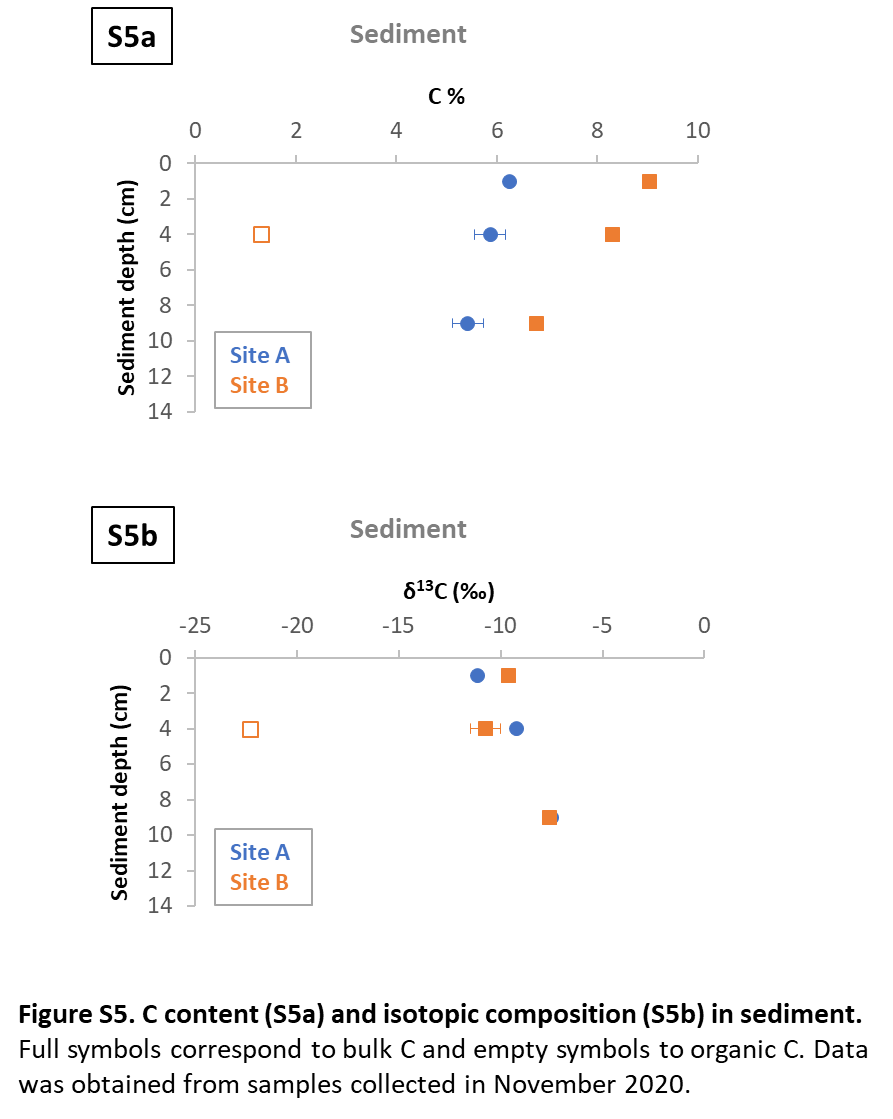


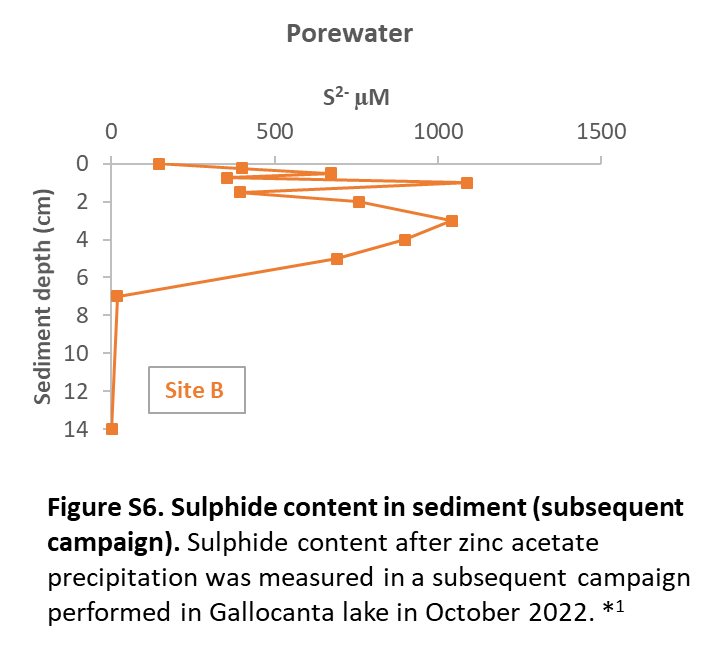


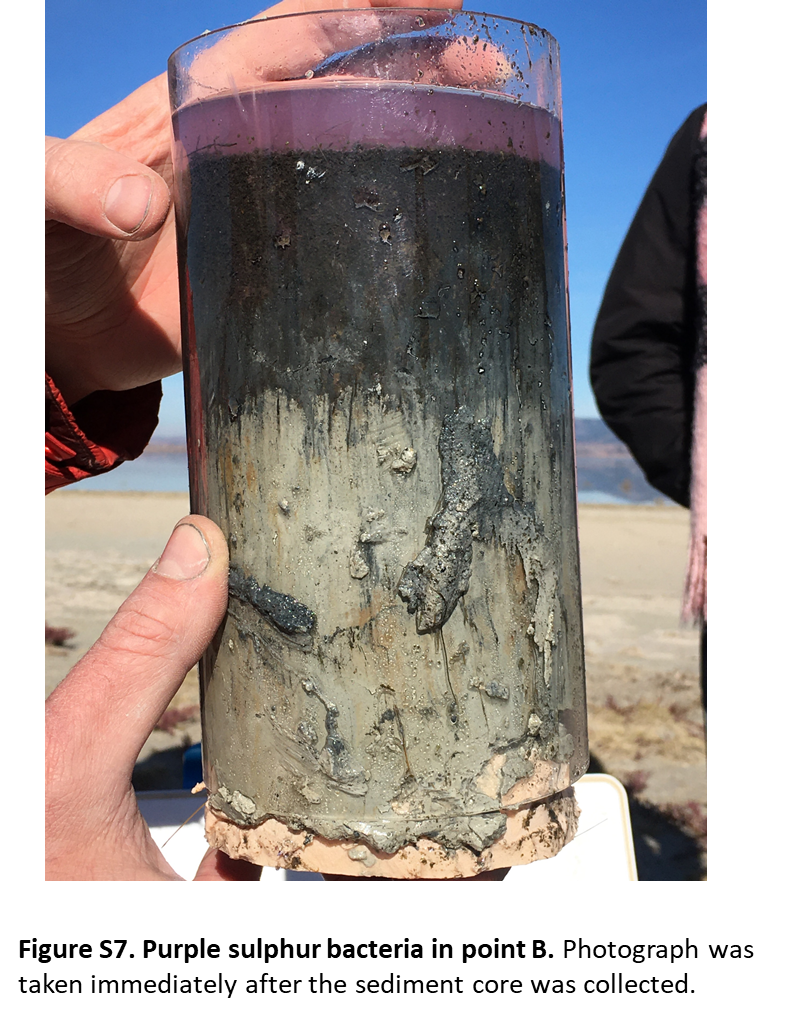


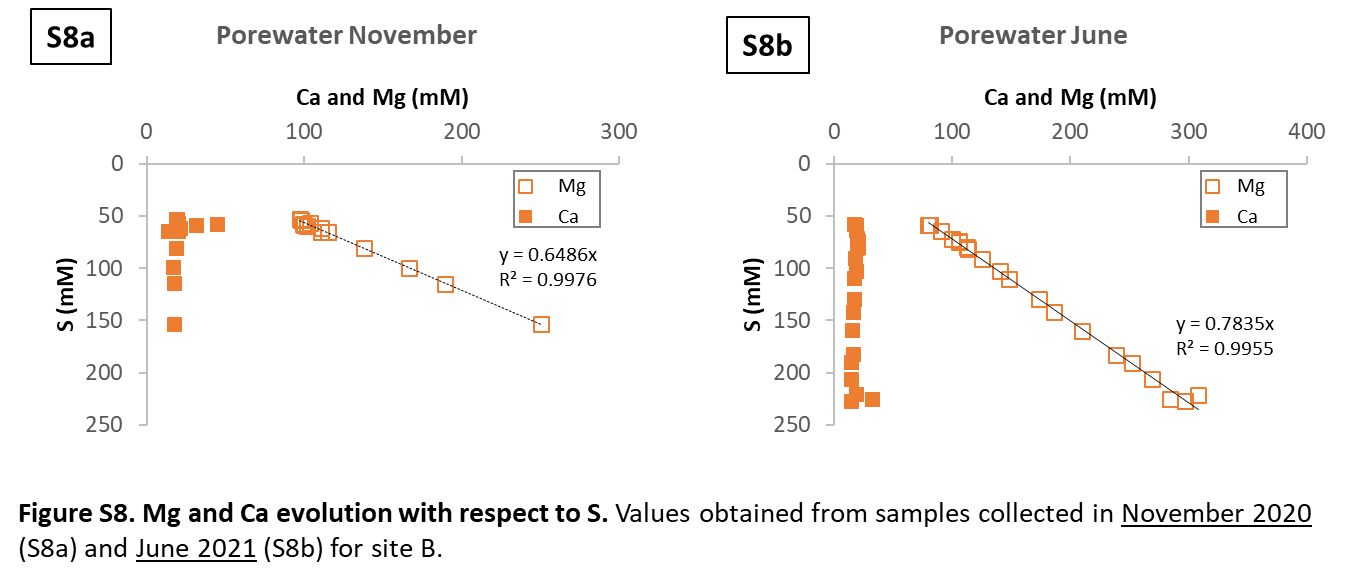


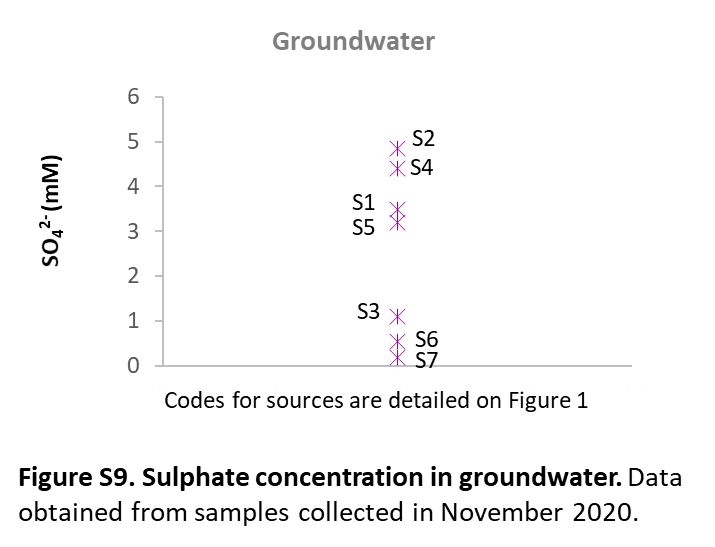


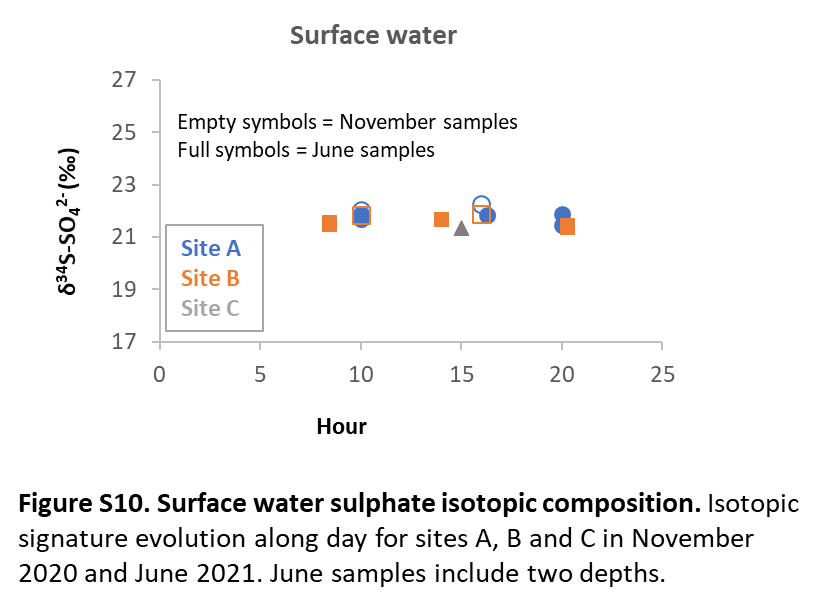


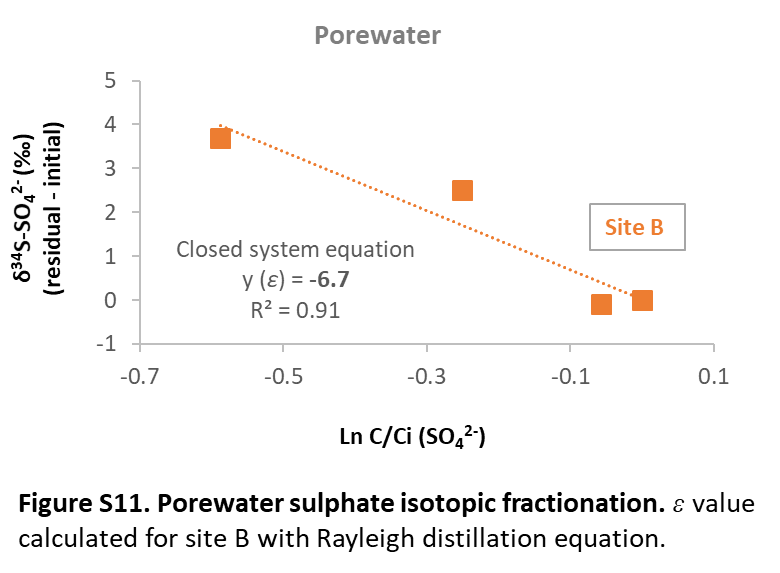


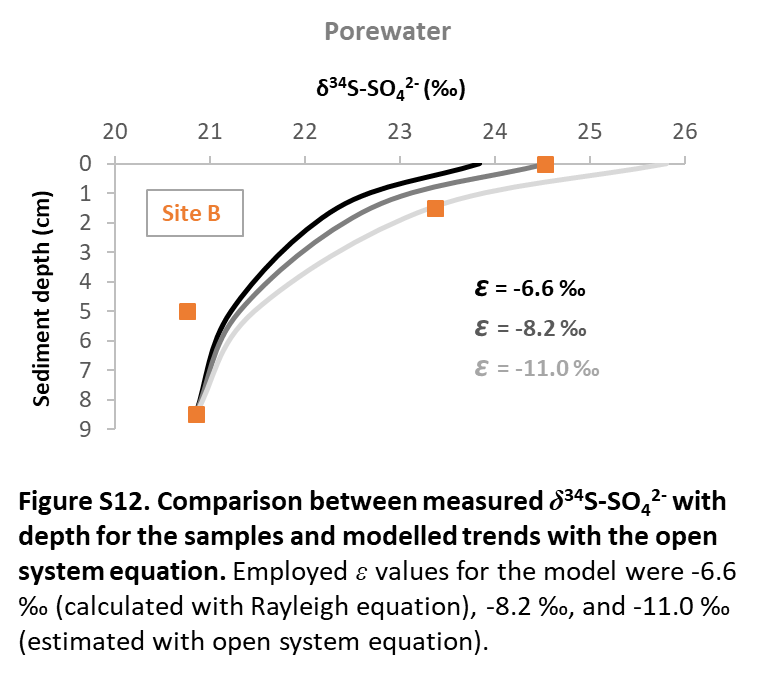


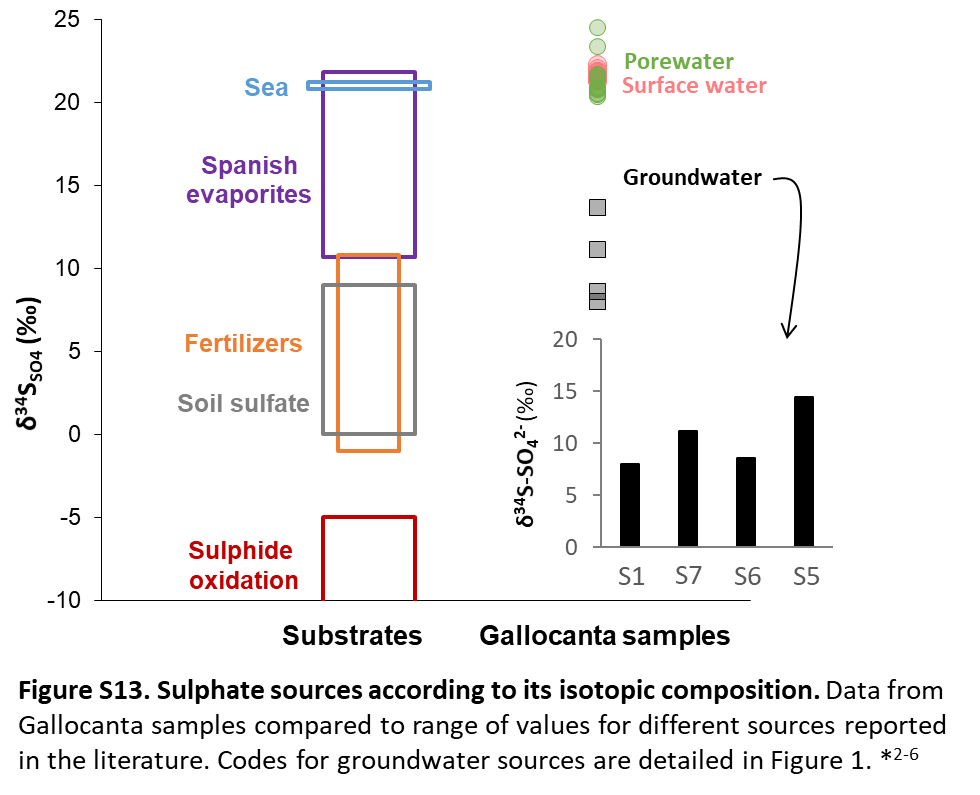


***REFERENCES**

1. Fonselius, S., Dyrssen, D. & Yhlen, B. Determination of hydrogen sulphide. In *Methods of Seawater Analysis* 91–100, John Wiley & Sons, Ltd (1999).

2. Clark, I. & Fritz, P. Environmental Isotopes in Hydrogeology. *Eos Trans. Am. Geophys. Union* **80**, 217–217 (1997).

3. Otero, N., Canals, À. & Soler, A. Using dual-isotope data to trace the origin and processes of dissolved sulphate: A case study in Calders stream (Llobregat basin, Spain). *Aquat. Geochem.* **13**, 109–126 (2007).

4. Otero, N. & Soler, A. Sulphur isotopes as tracers of the influence of potash mining in groundwater salinisation in the Llobregat Basin (NE Spain). *Water Res.* **36**, 3989–4000 (2002).

5. Utrilla, R., Pierre, C., Orti, F. & Pueyo, J. J. Oxygen and sulphur isotope compositions as indicators of the origin of Mesozoic and Cenozoic evaporites from Spain. *Chem. Geol.* **102**, 229–244 (1992).

6. Vitoria, L., Otero, N., Soler, A. & Canals, À. Fertilizer Characterization: Isotopic Data (N, S, O, C, and Sr). *Environ. Sci. Technol.* **38**, 3254–3262 (2004).
